# Isolation, Structural Characteristics Analysis of a *Vigna unguiculata* Polysaccharide VUP80-3 and Its Protective Effect on GES-1 Cells In Vitro

**DOI:** 10.3390/molecules28145566

**Published:** 2023-07-21

**Authors:** Yijun Fan, Erya Xu, Jie Ma, Xuebing Li, Yuanyuan Liu, Linlong Xu, Aoxue Luo

**Affiliations:** Department of Landscape Plants, Sichuan Agricultural University, Chengdu 611130, China

**Keywords:** *Vigna unguiculata*, polysaccharide, structural characterization, gastric mucosal cells

## Abstract

Cowpea (*Vigna unguiculata)* is one of the main edible legume vegetables in China, and it can improve spleen and stomach function. A polysaccharide component (VUP80-3) has been isolated from *V. unguiculata* in this study. The average molecular weight of VUP80-3 is 6.43 × 10^4^ Da, and the main monosaccharide group is glucose. The mass ratio of monosaccharide groups in the polysaccharide was glucose:galactose:arabinose:rhamnose:xylose:mannose:fucose = 152.36:24.50:16.53:8.13:1.26:0.97:0.82. NMR analysis showed that VUP80-3 has →4)-α-D-Galp (1→ and →4)-α-D-Glcp(1→ main chain and →3,4)-β-D-Glcp(1→, →4,6)-α-D-Glcp(1→ branch chains, and the terminal sugar is α-D-Glcp(1→. Biological activity test results showed that VUP80-3 at 1000 μg·mL^−1^ significantly increased the activity of ethanol injured GES-1 cells (*p* < 0.01) and significantly reduced reactive oxygen species (ROS) in ethanol injured GES-1 cells and inflammatory factors (IL-8, IL-1β and TNF-α,) in GES-1 cells. This compound also reduced the apoptosis rate (*p* < 0.05), thereby significantly reducing the oxidative damage caused by ethanol in GES-1 cells. Therefore, VUP80-3 is a potential drug to protect the gastric mucosa from damage.

## 1. Introduction

Approximately, 10% of the world’s people suffer from peptic ulcer diseases, of which gastric ulcer is the most common [1]. Alcohol can cause acute gastritis and damage to the gastric mucosa [2]. With the improvement of socioeconomic standards, alcohol consumption has increased significantly. Acute or chronic damage of the gastric mucosa caused by excessive or long-term alcohol consumption is increasing [3] and is becoming a main inducement of gastric mucosal damage. Gastric mucosal damage is the early pathological reaction of gastric ulcers. Therefore, prevention and treatment strategies for ethanol-induced gastric mucosal damage are key to mitigating alcohol stimulated gastric ulcers.

Polysaccharides are natural macromolecules with various biological activities [4]. These biomacromolecules have been used as adjunctive therapy for many diseases in recent years. Many natural polysaccharides have been proven to improve intestinal damage and increase antioxidant activity [5]. Therefore, some researchers have attempted to use bioactive polysaccharides to treat gastric ulcers. For example, Liu et al. [6] found in the rat model that Ginseng polysaccharide (GPS) can treat gastric ulcers by improving ulcer index (UI) and increasing levels of nitric oxide (NO) and prostaglandin E2 (PGE2) in gastric juice. Many researchers have found that, in the rat model, aloe polysaccharides can also prevent and treat gastric ulcers [7,8,9]. Fucopolysaccharide is also used to protect the gastric mucosa and treat gastric ulcers because of its role in improving the gastrointestinal tract. Wang et al. [10] showed that taking 100 mg·Kg^−1^ of fucoidan every day can effectively protect the gastric mucosa, prevent gastric mucosal damage, and have antioxidant and anti-inflammatory effects in the rat model. Kan et al. [11] also confirmed that the combination of fucoidan and wheat polypeptide can effectively reduce ethanol-stimulated gastric mucosal damage. *Hericium erinaceus* has been used as a food to improve gastrointestinal function in China, and researchers showed that the polysaccharide in the fruit body of *H. erinaceus* also showed an excellent protection effect on gastric mucosa [12]. Therefore, polysaccharides in plants or fungi that can improve gastrointestinal function have been the focus of extensive research. Nonetheless, these polysaccharides require further research.

*Vigna unguiculata* (*Vigna unguiculata* (L.) Walp) is a leguminous plant used as a main vegetable in China and a common staple food in northwest China. This vegetable can regulate and improve the function of the spleen and stomach, eliminate gastrointestinal inflammation, and prevent and treat gastrointestinal inflammation if consumed regularly. *V. unguiculata* is rich in nutrients, including proteins [13], polysaccharides [14], phenols [15], starch [16] and so on. Additionally, *V. unguiculata* has been considered to have many activities for many years, such as antioxidant [17], anti-aging, promoting intestinal peristalsis and regulating blood sugar [18]. These activities of *V. unguiculata* are very similar to some active polysaccharides. Therefore, we suspected that *V. unguiculata* polysaccharides may also have antioxidant and therapeutic effects on stomach diseases. Therefore, a new polysaccharide was isolated from *V. unguiculata* and its structure was analyzed in this research. Moreover, its protective effect against ethanol on gastric mucosa was investigated by establishing an ethanol-induced injury model of GES-1 cells. This study will provide a theoretical basis for the application and development of *V. unguiculata*.

## 2. Results

### 2.1. Preparation of V. unguiculata Polysaccharides

**Figure 1:**
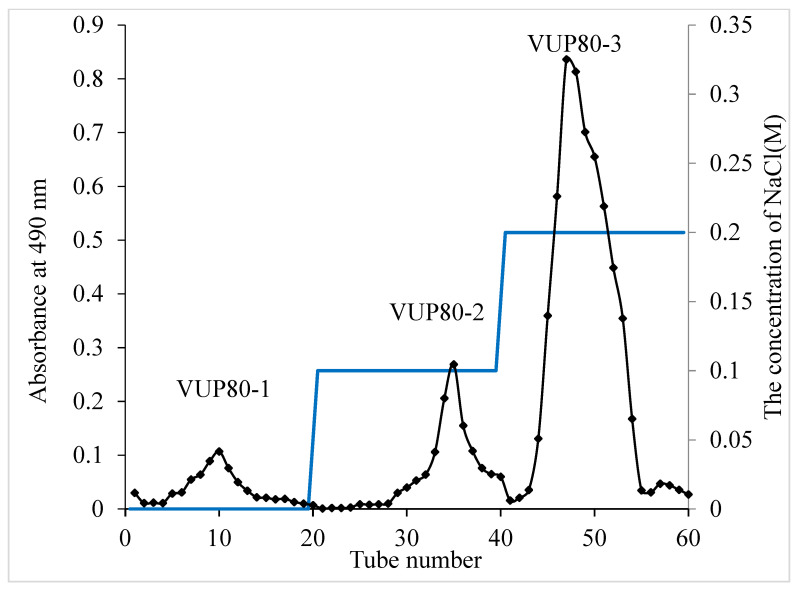
Chromatography of eluted crude polysaccharide (VUP80) on a DEAE-cellulose column (26 × 300 mm). VUP80-1 was eluted with deionized water, VUP80-2 was eluted with 0.1 M NaCl and VUP80-3 was eluted with 0.2 M NaCl.

Polysaccharides are a mixture; therefore, we used the ethanol segmented precipitation method to preliminarily separate *V. unguiculata* polysaccharides by molecular weight segments. In the preliminary experiment, we found that *V. unguiculata* crude polysaccharide VUP80 (precipitated by 80% ethanol, and the extraction rate was 7.59%) could protect the gastric mucosa. Therefore, we continued to separate and purify it again by using DEAE cellulose columns. After the aqueous solution of VUP80 was eluted with the DEAE cellulose column, three main peaks were observed (Figure 1) and named VUP80-1, VUP80-2 and VUP80-3, respectively. The mass ratios of the three polysaccharides obtained through DEAE cellulose column separation were: VUP80-1:VUP80-2:VUP80-3 = 5.17:10.64:84.19. Among them, VUP80-3 (eluted by 0.2 M NaCl) was present at the highest proportion and was the main polysaccharide. Therefore, we further investigated the structural characteristics and biological activity of VUP80-3. VUP80-3 was collected and freeze-dried until further use.

### 2.2. Molecular Weight of VUP80-3

After freeze-drying, a gray powder (VUP80-3) was obtained. The GPC analysis results showed that VUP80-3 exhibited a single peak. According to calculations based on the molecular weight standard curve (Figure S1), the molecular weight (Mw) of VUP80-3 was 6.43 × 10^4^ Da, which is within the middle range of the molecular weights reported for polysaccharides [19].

### 2.3. FTIR Spectra

**Figure 2:**
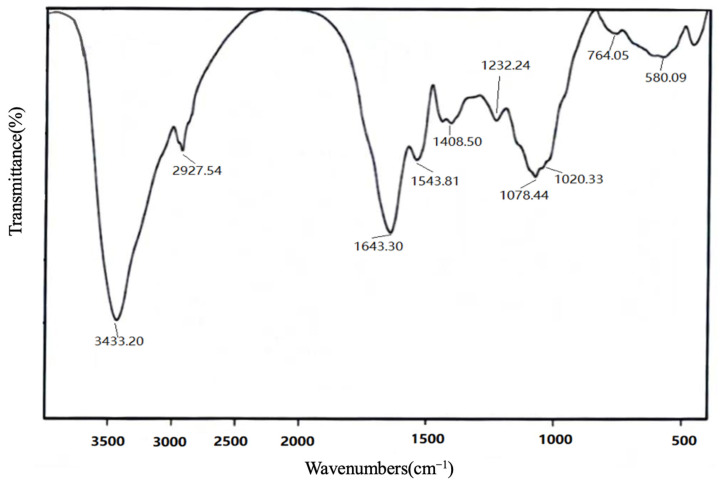
FTIR spectra of the polysaccharides of VUP80-3.

The IR of VUP80-3 is shown in Figure 2. The data showed that there was a wide peak at 3600–3200 cm^−1^ of sugars, which was the stretching vibration of the –OH in the sugar molecule, while C–H stretching and binding vibrations appeared at 3000–2800 cm^−1^ [20]. Additionally, the absorption peak at 1400–1200 cm^−1^ was the variable angle vibration of C–H [21]. As shown in Figure 2, VUP80-3 yielded a large absorption peak at 3433 cm^−1^, an absorption peak at 2927 cm^−1^ and an absorption peak at 1232 cm^−1^. These were characteristic peaks of carbohydrates [22], which showed that VUP80-3 was a carbohydrate compound. The absorption peak of 1665–1635 cm^−1^ was due to the bound water [23]. The absorption peaks at 1643 cm^−1^ and 1408.50 cm^−1^ were caused by the bending vibration of the C–H bond, and peaks at 1020 cm^−1^ and 1078 cm^−1^ were ascribed to the stretching vibration of C–O of C–O–C or C–O–H, which also indicates the existence of a pyran ring in VUP80-3 [24,25]. There was a characteristic peak of α-glycoside bond at 764 cm^−1^ [26].

### 2.4. Monosaccharide Analysis of VUP80-3

The monosaccharide composition of VUP80-3 was analyzed by Trifluoroacetic acid hydrolysis and ion chromatography. The results showed that there were seven main monosaccharide peaks in VUP80-3. Among them, the peak of glucose was the highest, indicating that glucose was the main monosaccharide group with the largest content in VUP80-3, followed by Galactose and Arabinose. According to the peak area calculation, the mass ratio of each monosaccharide group in the polysaccharide is as follows: glucose:galactose:arabinose:rhamnose:xylose:mannose:fucose = 152.36:24.50:16.53:8.13:1.26:0.97:0.82. Among them, glucose accounted for the highest proportion and was the main monosaccharide group of VUP80-3.

### 2.5. Methylation Analysis Results

Methylation analysis can provide detailed information of glycosidic bond types and detect monosaccharide units with less content. However, methylation analysis is not an independent analytic method. Therefore, it is often combined with other analysis methods to determine the glycosyl connection mode and location of the polysaccharide carbon chain [27].

According to the methylation test results, VUP80-3 was mainly composed of 10 carbohydrate units. The ratio of monosaccharide residues of methylation was consistent with the result of the monosaccharide analysis (for the TIC chromatogram see Figure S2). This indicates that the sugar chain was not decomposed during methylation (Table 1).

### 2.6. NMR Spectroscopy Analysis

**Figure 3:**
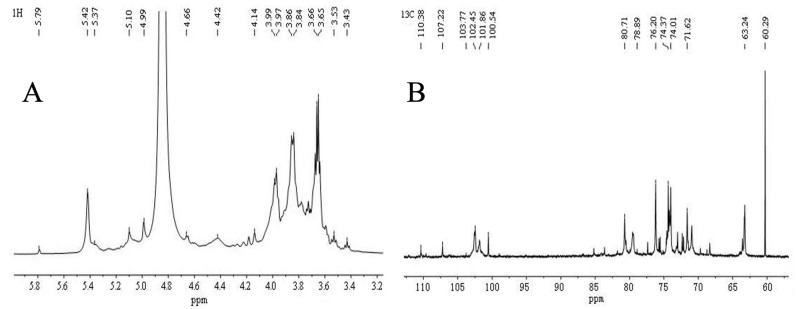

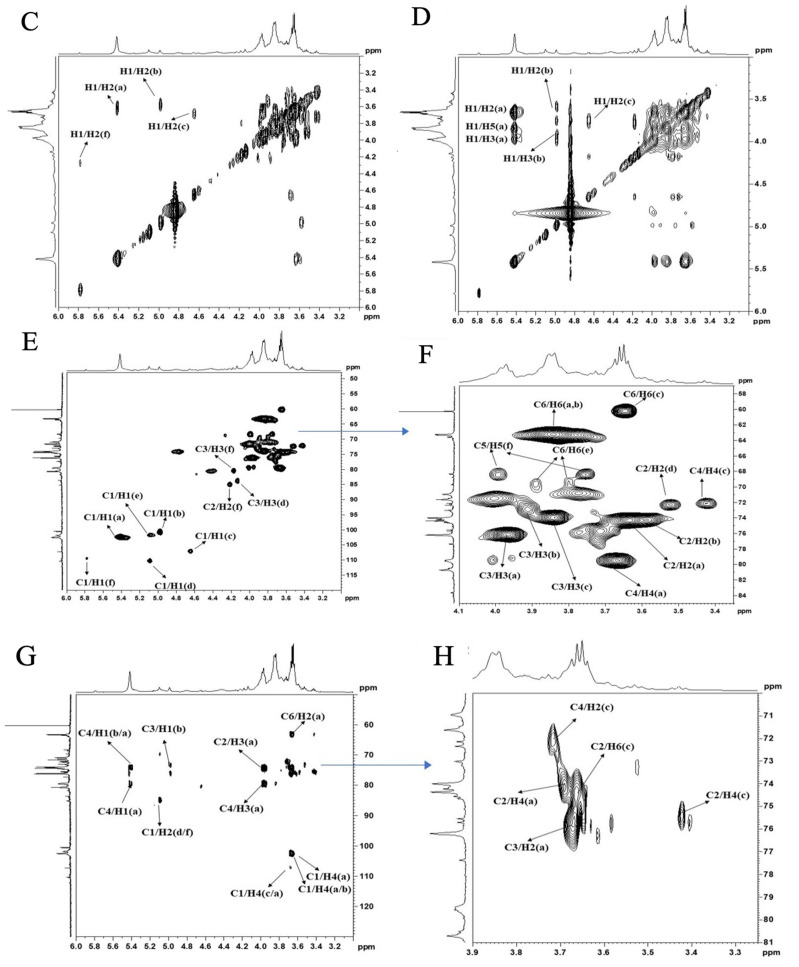
Nuclear magnetic resonance analysis of polysaccharide VUP80-3. (**A**) is ^1^H NMR spectra, (**B**) is ^13^C NMR spectra, (**C**) is COSY spectra, (**D**) is NOESY spectra, (**E**) is HSQC spectra, (**F**) is a partial enlarged view of HSQC spectra, (**G**) is HSQC spectra, and (**H**) is a partial enlarged view of HMBC spectra.

The polysaccharide signal in ^1^H NMR was concentrated at δ 3–6 ppm (Figure 3A,B). The β-anomeric hydrogen signals were distributed in δ 4.5–4.8 ppm, and α-anomeric hydrogen signals were distributed in δ 4.8–5.8 ppm. The ^1^H NMR signal of this sample was mainly distributed in δ 3.2–5.8 ppm. Six coupling signal peaks in the δ 4.5–5.8 ppm anomeric hydrogen signal region indicated six sugar residues. The non-anomeric hydrogen signals were concentrated in the δ 3.2–4.2 ppm area. Due to the serious overlap of individual signals, it was also necessary to assign the chemical shifts of H2–H6 of sugar residues A, B and C by combining the COSY and HSQC spectra.

As is shown in Figure 3, the anomeric carbon signals of VUP80-3 in ^13^C NMR were mainly concentrated at 95–110 ppm, and six coupling signal peaks were identified. Additionally, the chemical shifts were δ 110.36 ppm, δ 107.22 ppm, δ 103.77 ppm, δ 102.45 ppm, δ 101.86 ppm and δ 100.54 ppm (Figure 3B). In combination with the ^1^H NMR, COSY (Figure 3C), NOESY (Figure 3D), HSQC (Figure 3E,F) and ^13^C NMR spectra, each residue was assigned to determine the anomeric carbon signal of each residue. Based on the sample bonding structure (methylation) information, anomeric carbon signals, and comprehensive literature reports, residue A was determined as →4)-α-D-Glcp(1→, B was →4)-α-D-Galp(1→, C was α-D-Glcp(1→, D was →3,4)-β-D-Glcp(1→, E was →4,6)-α-D-Glcp(1→, F was →5)-α-L-Araf(1→ and assigned its ^1^H and ^13^C chemical shifts (Table 2).

Based on Figure 3 and Table 2, we analyzed the possible structures and connection mode of UVP80-3. We found a coupling signal between H1 (5.42 ppm) and C4 (79.47 ppm) of sugar residue A, C1 (102.45 ppm) and H4 (3.68 ppm) of sugar residue A, and H4 (36.5 ppm) of sugar residue B, as well as C1 (107.22 ppm) of sugar residue C and H4 (3.69 ppm) of sugar residue A. We also found that there was a cross peak between H1 and H4 of residue A and H4 of residue B. From this, we inferred that the main chain of UVP80-3 may be “→4)-α-D-Glcp(1→” and “→4)-α-D-Galp(1→”, while branched chains may be “→3,4)-β-D-Glcp(1→”, “→4,6)-α-D-Glcp(1→” and “α-D-Glcp(1→”.

### 2.7. SEM Analysis

**Figure 4:**
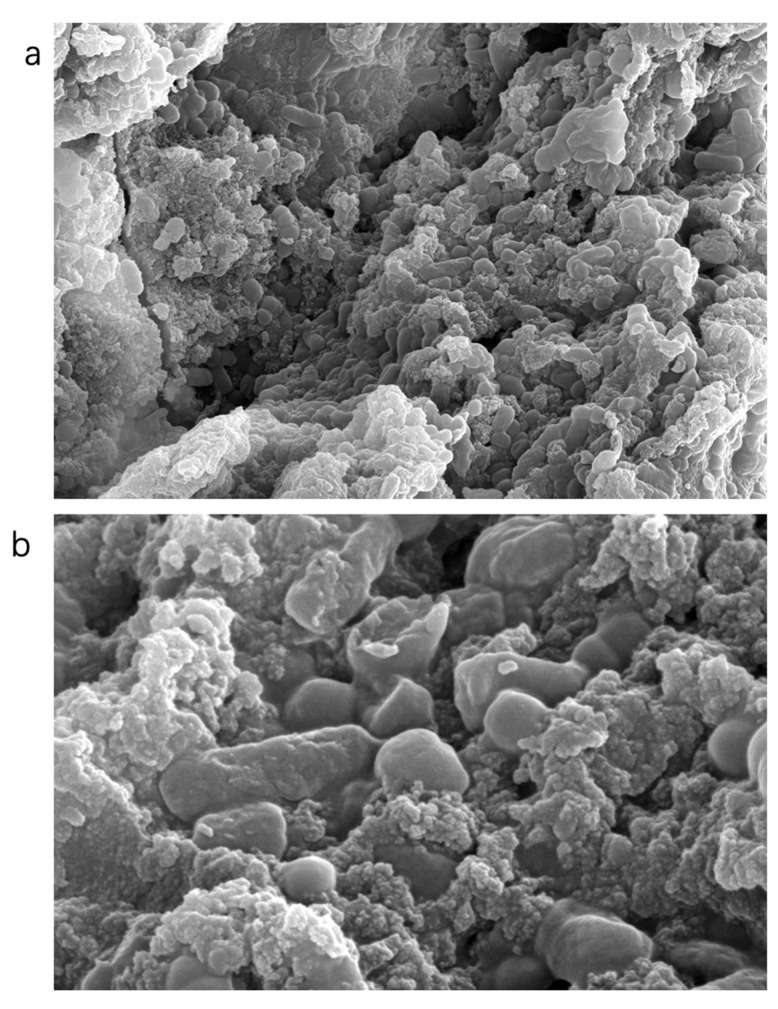
SEM analysis results of polysaccharide VUP80-3 (**a**) at a magnification of 5000× and a resolution of 10 μm and (**b**) at a magnification of 20,000× and a resolution of 3 μm.

At 5000× magnification (Figure 4) the surface of the polysaccharide VUP80-3 presented a mass or clastic accumulation, and the surface was uneven with small grain folds. At 20,000× magnification, the surface of VUP80-3 presented two different morphologies, some of which were cylindrical particles with smooth surfaces and others which were semicircular particles with regular structures and dispersed molecules. Certain polysaccharides present clastic accumulation, with uneven surfaces and irregular geometric shapes. This showed that there was a repulsive force between polysaccharide molecules, and the intermolecular attraction was small [28].

### 2.8. Effect of VUP80-3 on the Activity of Ethanol-Induced GES-1 Damaged Cells

*V. unguiculata* can improve spleen and stomach function and eliminate gastrointestinal inflammation. Regular consumption can prevent and treat gastrointestinal inflammation [29]. *V. unguiculata* is rich in polysaccharides, which are also considered active ingredients which protect the gastric mucosa. However, it is not clear how these polysaccharides protect the stomach. Therefore, we preliminarily explored the protective effect of soybean polysaccharide VUP80-3 on injured GES-1 cells.

#### 2.8.1. Establishment of GES-1 Cell Injury Model

**Figure 5:**
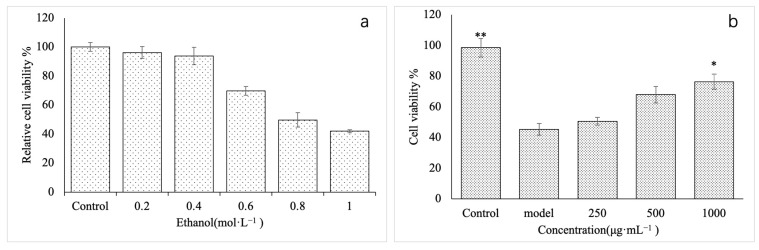
Establishment of GES-1 damaged cell model. (**a**) is the damage of different concentrations of ethanol to GES-1 cells, and (**b**) is the effect of different concentrations of polysaccharide VUP80-3 on the viability of ethanol-induced GES-1 damaged cells. * *p* < 0.05 and ** *p* < 0.01 when compared with the model group.

First, we established a model of ethanol damage on GES-1 cells and analyzed its effect on the proliferation of these cells. GES-1 cells were treated with different concentrations of ethanol. After 8 h of treatment, the proliferation activity of GES-1 cells was detected using the CCK-8 method. As shown in Figure 5a, the low ethanol concentration had no significant effect on the proliferation activity of cells (*p* > 0.05). With the increase in ethanol concentration, the proliferation activity of GES-1 cells showed a significant downward trend. At 0.8 mol·L^−1^, proliferation activity was only 49.7% of the control, and compared with the control group, the difference was significant (*p* < 0.05). When the concentration reached 1.0 mol·L^−1^, the proliferation activity of GES-1 cells was 41.98%, and there was no significant difference compared with the activity at 0.8 mol·L^−1^ (*p* > 0.05). Therefore, 0.8mol·L^−1^ ethanol was used to establish the GES-1 cell injury model.

In order to investigate the effect of VUP80-3 on the viability of GES-1 cells injured by ethanol, we used three VUP80-3 concentrations, namely a low (250 μg·mL^−1^), medium (500 μg·mL^−1^) and high concentration (1000 μg·mL^−1^). Additionally, the control group (untreated normal cells) and the model group (ethanol injury group not treated with VUP80-3) were used for comparison. The proliferative activity of the model group was significantly lower than the control (Figure 5b) (*p* < 0.05). Compared with the model group, each treatment group exhibited increased activity in damaged GES-1 cells at varying degrees. Moreover, the improvement observed in the treated groups was concentration dependent. Among them, the activity of GES-1 cells of the high treatment group (1000 μg·mL^−1^) was significantly higher than that in the model group (*p* < 0.01).

#### 2.8.2. Effect of Polysaccharide VUP80-3 on ROS in Injured GES-1 Cells

Studies have shown that free radicals play an important role in ethanol-induced gastric mucosal injury. The levels of lipid peroxides and free radicals in the gastric mucosa after chronic alcohol consumption are very high [30]. The reason may be that ethanol can promote excessive production of ROS. ROS can directly damage the capillary endothelial cells of the gastric mucosa, and depletion of mucosal antioxidant defense, leading to excessive formation of lipid peroxide Glutathione peroxidase (GPx) and its substrate, reduced glutathione (GSH) [31]. Moreover, it can promote macrophages and multinucleated leukocytes to adsorb on the surface of endothelial cells, causing plasma exudation and bleeding. This reduces the blood flow of the gastric mucosa, leading to reduced secretion of mucus and bicarbonate and to H+ reflux. H+ regurgitation can also damage the vascular system of the mucosa, causing bleeding in the stomach cavity and aggravating the degree of tissue ischemia. Concomitantly, due to the change in pH in the mucosa, xanthine oxidase persists, producing oxygen free radicals continuously and further aggravating the tissue damage [32]. ROS play a significant role in ethanol-induced gastric mucosal injury.

**Figure 6:**
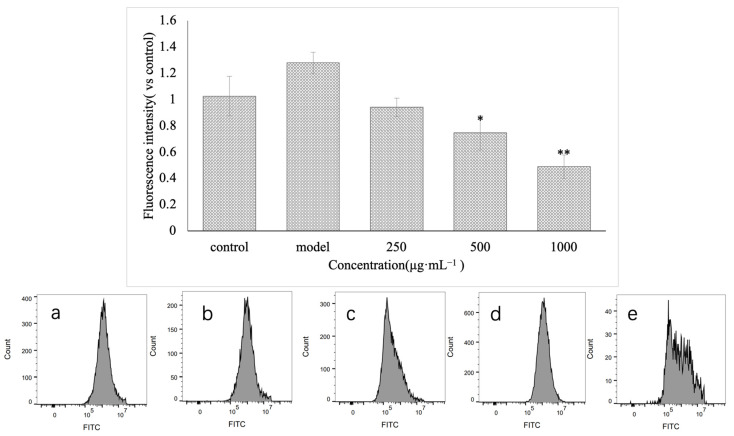
The effect of polysaccharides on ROS in cells injured by GES-1. (**a**) is the low polysaccharide concentration group (250 μg·mL^−1^), (**b**) is the polysaccharide medium concentration group (500 μg·mL^−1^), (**c**) is the polysaccharide high concentration group (1000 μg·mL^−1^), (**d**) is the control group, and (**e**) is the model group. * *p* < 0.05 and ** *p* < 0.01 when compared with the model group.

This experiment detected the change in ROS in injured GES-1 cells before and after treatment with VUP80-3. Additionally, the control group and the model group were set. As shown in Figure 6, the concentration of ROS in GES-1 cells treated with ethanol increased significantly compared with that in the control group (*p* < 0.05). Nonetheless, each group treated with VUP80-3 exhibited decreased ROS content in a concentration-dependent manner. At medium (500 μg·mL^−1^) and high concentrations (1000 μg·mL^−1^), the polysaccharide VUP80-3 significantly reduced the ROS content in the injured cells (41.6% and 61.7% lower, respectively; *p* < 0.05) compared with that in the model group. These data demonstrated that the polysaccharide VUP80-3 can effectively reduce ROS in ethanol damaged GES-1 cells, thereby alleviating the damage to these cells.

#### 2.8.3. Effect of VUP80-3 on the Secretion of Inflammatory Factors by Injured GES-1 Cells

Gastric mucosal injury can activate the immune system in the body leading to the activation of various inflammatory cells, recruitment to inflammatory sites and secretion of a variety of inflammatory cytokines [33]. Therefore, gastric mucosal injury leads to the inflammatory reaction of gastric mucosal tissue. TNF-α is a cell regulatory protein with important biological functions and is produced by the body under external stimulation. Additionally, it is considered an inflammatory protein, closely related to injury. After ethanol damage to the gastric mucosa, TNF-α expression was significantly increased. IL-1β can stimulate various immune and inflammatory cells, promote the release of inflammatory proteins and mediators, have a chemotactic effect on neutrophils, macrophages, and lymphocytes, and enhance the inflammatory response [34]. After ethanol damage to the gastric mucosa, the immune inflammatory factor IL-1β will also increase significantly [35]. However, as a neutrophil chemotactic and activating factor, IL-8 can activate neutrophils to cause a local inflammatory reaction and gastric mucosal damage [36]. Studies have suggested that when the parietal cells secrete less H+, the inflammatory response is weakened and the expression of nuclear factor (NF)-kB is gradually reduced, further reducing inflammatory cytokines such as TNF-α and IL-8. In turn, the inflammatory cells will gradually reduce, and the ulcer will gradually heal [37]. Therefore, drugs that interfere with the production of inflammatory factors may reduce the damage to the gastric mucosa. Considering this, we examined the secretion of inflammatory factors by injured GES-1 cells treated with polysaccharide VUP80-3 in vitro.

The results (Table 3) showed that the level of inflammatory factors in the model group was significantly higher than that in the control group (*p* < 0.01), indicating that ethanol treatment caused damage to GES-1 cells leading to increased IL-8, TNF-α and IL-1β cytokine secretion by inflammatory cells. Compared with the model group, the VUP80-3 treatment groups exhibited decreased levels of inflammatory factors. At a high concentration (1000 μg·mL^−1^), VUP80-3 significantly reduced the levels of TNF-α (*p* < 0.01), IL-1β (*p* < 0.05) and IL-8 (*p* < 0.01) in injured cells. These data indicate that VUP80-3 at 1000 μg·mL^−1^ can inhibit the expression of gastric mucosal inflammatory factors, thus preventing further damage to the gastric mucosa by these inflammatory cytokines and enabling the repair of the gastric mucosa.

#### 2.8.4. Effect of Polysaccharide VUP80-3 on Apoptosis of Injured GES-1 Cells

Research shows that the pathogenesis of ethanol-induced gastric injury involves mechanisms other than oxidative stress and gastritis [38]. ROS trigger apoptosis in the gastric mucosa, which also plays an important role in gastric mucosal injury. Therefore, reducing apoptosis is an effective way for cells to resist oxidative damage. To verify whether the resistance provided by VUP80-3 to cell oxidation is related to the reduction in apoptosis, flow cytometry was used to analyze cell changes in each treatment group.

**Figure 7:**
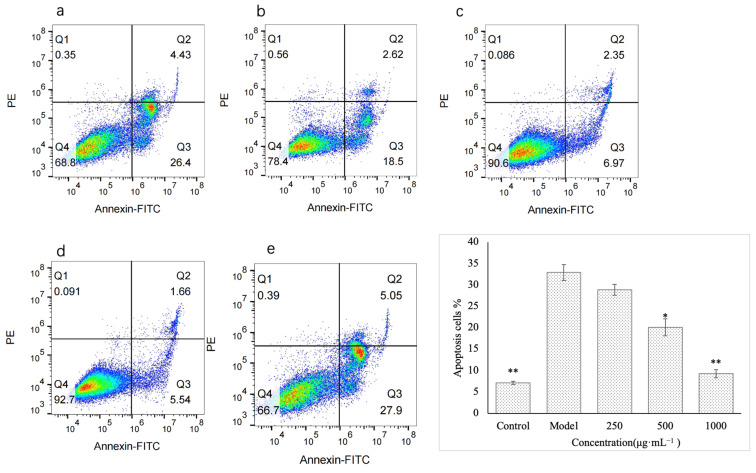
The effect of polysaccharides on apoptosis induced by GES-1. (**a**) is the low polysaccharide concentration group (250 μg·mL^−1^), (**b**) is the polysaccharide medium concentration group (500 μg·mL^−1^), (**c**) is the polysaccharide high concentration group (1000 μg·mL^−1^), (**d**) is the control group, and (**e**) is the model group. * *p* < 0.05 and ** *p* < 0.01 when compared with the model group.

The flow cytometry results are shown in Figure 7. Compared with the control group, the apoptosis rate of GES-1 cells in the model group was significantly increased (*p* < 0.01). This indicated that ethanol treatment caused apoptosis in gastric mucosal epidermal cells, which may also be an important reason for ethanol-induced gastric mucosal damage. The level of apoptosis in each polysaccharide treatment group decreased. However, the low concentration (250 μg·mL^−1^) had no significant effect on the apoptosis level of injured GES-1 cells. With the increase in polysaccharide concentration, the apoptosis level of cells decreased gradually. The medium concentration group (500 μg·mL^−1^) exhibited 38.9% less apoptosis than the model group (*p* < 0.05). At the high concentration (1000 μg·mL^−1^), the level of apoptosis in the polysaccharide treatment group was the lowest (71.7% lower) compared to that in the model group. Compared with the model group, there was a very significant difference (*p* < 0.01), and compared with the control group, there was no significant difference in the level of apoptosis. The results showed that VUP80-3 could significantly reduce the apoptosis rate of injured GES-1 cells at 1000 μg·mL^−1^ and restore it to the level of normal cells. Therefore, reducing apoptosis is another way for VUP80-3 to protect the damaged GES-1 cells and enable the repair of gastric mucosal damage. This result may also be related to the extremely significant reduction of the ROS level in the injured GES-1 cells in the high concentration group.

## 3. Discussion

The incidence rate of acute or chronic gastritis and gastric ulcer due to the damage of gastric mucosal epithelium caused by drinking has been rising, which has become a serious problem affecting health in the drinking culture [39]. Therefore, protecting the gastric mucosa is key to treating these diseases and is a major clinical challenge. At present, the drugs used to treat gastric mucosal injury mainly include antacids, proton pump inhibitors and histamine H2 receptor antagonists. The main mechanism of these drugs is to achieve gastric mucosal protection by inhibiting gastric acid secretion, neutralizing gastric acid, inhibiting the hydrogen potassium ATPase on the parietal cells of gastric mucosa, or selectively binding to the histamine H2 receptor and competitively antagonizing the effect of histamine on the H2 receptor, thereby inhibiting gastric acid secretion [40]. However, these drugs can cause side-effects that cannot be ignored, such as hepatitis, nephritis, osteoporotic fracture and gastrin [32]. Therefore, exploring new therapeutic targets and finding safe and effective drugs for gastric mucosal protection were important tasks in the treatment of gastric diseases. In this study, we found that 1000 μg·mL^−1^ of VUP80-3 can significantly increase the vitality of ethanol damaged GES-1 cells (*p* < 0.01), significantly reduce the levels of ROS and inflammatory factors in ethanol damaged GES-1 cells (*p* < 0.05), and also reduce the rate of cell apoptosis (*p* < 0.05), significantly reducing the oxidative damage of GES-1 cells caused by ethanol, thus achieving the goal of repairing damaged gastric mucosa cells. Therefore, it can be speculated that VUP80-3 may reduce cell apoptosis rate by reducing ROS and inflammatory factors, thereby repairing the gastric mucosa. The principle of action was different from existing gastric mucosal protective drugs. Therefore, this study may provide new ideas for drug research to protect gastric mucosal damage.

With the continuous improvement of food science technology and medical level as well as the change in people’s diet concepts, bean food is becoming more and more popular. Legumes include soybeans (*Glycine max*), red beans (*Vigna angularis*), mung beans (*Vigna radiata*), black beans (*Glycine max*), lentils (*Lablab purpureus*), cowpeas (*Vigna unguiculata*), kidney beans (*Phaseolus vulgaris*), etc. Polysaccharides are a type of natural polymer compounds that, together with nucleic acids, proteins, etc., determine the functionality of species [41]. In recent years, plant polysaccharides have become a research hotspot due to their wide sources and important biological activities [42]. Legume polysaccharides belong to plant polysaccharides, and as one of the active components of beans, they have gradually attracted widespread attention. For example, glycine max polysaccharide can promote the generation of myelocytes in bone tissue, stimulate the production of hematopoietic growth factors in splenocyte, and also promote the gene expression of neutrophil colony-stimulating factor [43]. Wu et al. [44] studied the effects of black bean water-soluble polysaccharide on the expression of granulocyte colony-stimulating factor in human blood. The results showed that black bean polysaccharide could induce the expression of granulocyte colony-stimulating factor. *Lablab purpureus* polysaccharides played an important role in antioxidant and immune functions, as well as in protecting neuronal cells from apoptosis. Hu et al. [45] found that lentil polysaccharides (WHBP) can stimulate a significant increase in nerve cell viability and also resist hypoxic neuronal apoptosis. Bean polysaccharides have a certain preventive effect on colon cancer [46].

In recent years, research on legumes has mostly focused on common legume varieties such as soybeans and mung beans, while research on cowpeas has only just begun. For example, Cheng et al. [47] found that the scavenging effect of cowpea bound extract on FRAP, ABTS and oxygen free radicals was significantly higher than that of free extract, and the scavenging effect of free extract on DPPH free radicals was also significantly higher than that of bound extract. Cowpea polysaccharide can significantly improve the glycogen content, FBG, TG, TC, LDL-C, ISI and the number of islet cells in diabetes mice [48]. Lai et al. [49] isolated and purified two acidic polysaccharide components, MP1 and MP2, from cowpea skin. Both were heteropolysaccharides, containing 9.9% and 36.4% aldehyde acids, respectively. MP1 is composed of mannose with a molecular weight of 83 kDa, and MP2 is composed of rhamnose and galactose with a molecular weight of 45 kDa. Yang et al. [50] used ultrasonic assistive technology to prepare cowpea total polysaccharide, which can eliminate DPPH free radicals and ABTS free radicals. In this study, we obtained a new cowpea polysaccharide VUP80-3 by ethanol fractionation precipitation combined with ion exchange resin separation. This polysaccharide was different from the reported cowpea polysaccharide, which was mainly composed of glucose, galactose, arabinose, rhamnose, xylose, mannose and fucose, did not contain uronic acid, and its molecular weight was 6.43 × 10^4^ Da. Due to the structural characteristics (monosaccharide composition, molecular weight and main chain) of VUP80-3 being different from the previously reported cowpea polysaccharides, there were significant differences in its activity. This study found for the first time that cowpea polysaccharide VUP80-3 has a protective effect on ethanol-induced gastric mucosal damage, and there have been no other relevant reports so far.

## 4. Materials and Methods

### 4.1. Materials and Reagents

*V. unguiculata* was obtained from Chengdu (Sichuan province, China; 30°05′ N–31°26′ N, 102°54′ E–104°53′ E). The whole cowpea harvested in September 2022 was used as the experimental material, and the cowpea was dried and crushed for use. Standard monosaccharides were purchased from the China Institute for the Control of Pharmaceutical and Biological Products (Beijing, China). Roswell Park Memorial Institute (RPMI)-1640 medium was purchased from Hyclone in the USA. Pancreatin and penicillin were purchased from Wisent. The GES-1cell line was purchased from the cell bank of the Chinese Academy of Sciences (Shanghai, China). TNF-α, IL-8 and IL-1β kits were purchased from Nanjing Jiancheng Bioengineering Company (Nanjing, China). All other reagents were analytical pure reagents (Kelong Chemical Reagent Co., Chengdu, China).

### 4.2. Preparation of V. unguiculata Polysaccharide

The *V. unguiculata* polysaccharide was obtained using the improved method of Fan et al. [51]. Firstly, *V. Unguiculata* was dried at 60 °C to a constant weight, crushed, and passed through a 40 mesh sieve. A total of 50 g of *V. unguiculata* powder was sequentially refluxed with 500 mL petroleum ether and 500 mL anhydrous ethanol, which was then filtered. The extraction was repeated three times and the filtrate was discarded. Then, 500 mL of distilled water was added to the filter residue for reflux extraction for 2 h; this was filtered, and the filtrate was collected. The Sevag reagent (n-butanol:chloroform = 1:4) extraction method was used to remove proteins from the polysaccharide. Next, the extraction solution was accurately measured, anhydrous ethanol was added until the ethanol content in the system reached 60%, the solution was filtered, and the precipitate was discarded. Anhydrous ethanol was continuously added to the filtrate until the ethanol content in the system was 80%; then the solution was filtered, and the precipitate was collected. Then the precipitation was dialyzed by using a dialysis membrane (7000 Da) for 48 h, and a crude polysaccharide sample was obtained, freeze-dried and labeled as C-VUP80. C-VUP80 was dissolved in deionized water and purified by AB-8 (Polystyrene-type weakly polar adsorption resin) and ADS-7 (Macroporous adsorption resin) columns to remove residual pigments and proteins. Then the effluent was collected and freeze-dried to obtain the VUP80 polysaccharide.

A total of 1.0 g of VUP80 was dissolved in 30 mL of ddH_2_O, then separated by DEAE-cellulose column (2.6 × 30 cm). Elution conditions were as follows: Elution solution: ddH_2_O, 0.1 M NaCl and 0.2 M NaCl, at a flow rate of 1.0 mL·min^−1^ at room temperature. A fully automatic partial collector was used to collect sewage at a rate of 8.0 mL·Tub^−1^. The absorbance value of the effluent was measured at 490 nm by using the phenol sulfuric acid method.

### 4.3. Molecular Weight Analysis

The molecular weight of the polysaccharide was determined by high performance liquid gel permeation chromatography (GPC) (Waters 515, Waters Co Ltd., Milford, CT, USA). The detection conditions were as follows: the chromatographic column was a Waters ultrahydrogel column (300 × 7.8 mm), room temperature, eluent: 0.2M phosphate buffer (pH 7.0), flow rate: 0.70 mL·min^−1^. The standard sample was dextran (molecular weights: 2500, 4600, 7100, 10,000, 21,400, 41,100, 84,400, 133,800 and 200,000 Da) with a sample size of 10 μL, and the sample concentration was 5 mg·mL^−1^ [52].

### 4.4. Infrared Spectrum Analysis

A total of 1 mg of the polysaccharide sample was mixed with KBr, which was then pressed into a sheet, and scanning analysis was performed at 4000 cm^−1^–500 cm^−1^ using a Nicolet6700 (Thermo Fisher Scientific, Waltham, MA, USA) Fourier transform infrared spectrometer [53].

### 4.5. Monosaccharide Composition Analysis

A polysaccharide sample of 5.0 mg ± 0.05 mg was accurately weighed, and then 1.0 mL of 2 M TFA solution was added and hydrolyzed at 121 °C for 2 h. The solvent was dried with nitrogen and cleaned 2–3 times with methanol. ddH_2_O was added to dissolve the hydrolysate and was transferred to a chromatographic flask for testing. The chromatography system was the Thermo ICS5000 ion chromatography system (ICS5000, Thermo Fisher Scientific, Waltham, MA, USA), which used an electrochemical detector to analyze and detect monosaccharide components. At the same time, quantitative analysis of each monosaccharide was conducted using the external standard method.

Adopting Dionex ™ CarboPac ™ PA20 (150 × 3.0 mm, 10 μm) chromatography column. The injection volume was 5.0 μL. Mobile phase A was 0.1 M NaOH, mobile phase B was 0.1 M NaOH and 0.2 M NaAc, and flow rate was 0.5 mL/min. The column temperature was 30 °C. Elution gradient: 0min A phase/B phase (95:5 V/V), 30 min A phase/B phase (80:20 V/V), 30.1min A phase/B phase (60:40 V/V), 45min A phase/B phase (60:40 V/V), 45.1 min A phase/B phase (95:5 V/V), and 60 min A phase/B phase (95:5 V/V). Chromatographic data was processed using Chromeleon software [54,55].

### 4.6. Methylation Analysis

Methylation analysis of the polysaccharide sample was performed using gas chromatography-mass spectrometry (GC-MS). The polysaccharide sample was dissolved in 500 μL of dimethyl sulfoxide (DMSO), then 1.0 mg of NaOH was added and the sample was incubated for 30 min. After incubation, 50 μL iodomethane solution was added for 1 h. After the reaction was completed, 1.0 mL H_2_O and 2.0 mL of dichloromethane were added and mixed evenly with a vortex. The mixture was centrifuged, and the aqueous phase was discarded. Taken a sample of a certain concentration, and added 2M TFA to the sample and reacted at 121 °C for 90 min. The sample was allowed to dry at 30 °C. An amount of 50 μL ammonia (2 M) and 50 μL NaBD4 (1 M) were added to the evaporated sample, which was thoroughly mixed and reacted at room temperature for 2.5 h. Subsequently, we added 20 μL acetic acid to stop the reaction. After being dried by nitrogen, acetic anhydride (250 μL) was added, and the solution was vortexed and incubated. We then added 500 μL dichloromethane followed by vortex centrifugation and discarding of the aqueous phase. The lower dichloromethane phase was passed through a 0.45 μM filter membrane for on-line detection [56].

The Agilent gas chromatographic system (Agilent 7890A, Agilent Technologies, Santa Clara, CA, USA) was employed. The chromatographic conditions were as follows: BPX70 (30 m × 0.25 mm × 0.25 µm, SGE, Australia), injection: 1 μL, split ratio: 10:1, carrier gas: high-purity helium, temperature of column: 140 °C for 2.0 min, 230 °C at 3 °C ·min^−1^ and maintained for 3 min.

The mass spectrometry system uses Aiglent’s quadrupole mass spectrometry detection system (Agilent 5977B; Agilent Technologies, USA), equipped with an electron bombardment ion source (EI) and a MassHunter workstation. The analyte is detected in full scan (SCAN) mode, with a quality scanning range (*m/z*) of 30–600.

### 4.7. NMR Spectroscopy Analysis

The purified polysaccharide was fully dissolved in D_2_O to prepare a 40 mg·mL^−1^ polysaccharide solution. The solution (0.5 mL) was placed in a nuclear magnetic tube and the nuclear magnetic resonance spectrometer (Bruker 600 MHz) was used to scan the ^1^H and ^13^C spectra of one-dimensional nuclear magnetic resonance, as well as the COSY, HSQC, HMBC and NOESY spectra of two-dimensional nuclear magnetic resonance [57].

### 4.8. Scanning Electron Microscopy (SEM) Analysis

After coating the dried polysaccharide with 25 nm thickness ion sputtering (Pt/At powder), the sample was observed at a potential of 5 kV under a vacuum condition by a scanning electron microscope (QuantaTM FEG SEM, Stoney Creek, NC, USA). The magnification range was from 5000 to 20,000 times [58].

### 4.9. Protection of VUP80-3 against Ethanol-Induced GES-1 Injury

#### 4.9.1. Cell Culture and Establishment of GES-1 Cell Injury Model

GES-1 was cultured in RPMI-1640 culture medium containing 10% FBS under conditions of 37 °C, saturated humidity, and 5% CO_2_ concentration. Logarithmic growth GES-1 with different concentrations of ethanol (0.2M, 0.4 M, 0.6 M, 0.8 M and 1.0 M) were added to the cells and cultured for 8 h. The proliferative activity of GES-1 cells was detected to determine the ethanol concentration used to establish a GES-1 cell injury model [3].

#### 4.9.2. Analysis of Intracellular ROS and Inflammatory Factors

The experimental groups were as follows: control group (normal GES-1 cell group), model group (ethanol treatment), low polysaccharide concentration group (250 μg·mL^−1^ + EtOH), medium polysaccharide concentration group (500 μg·mL^−1^ + EtOH) and high polysaccharide concentration group (1000 μg·mL^−1^ + EtOH). Cells were digested routinely and counted to obtain 5.0 × 10^4^ cells·well^−1^ in a 96-well plate, 100 cells·μL^−1^. After 8 h of cultivation, the supernatant was discarded. Then 20 μL CCK-8 solution was added to each well. After continuing to cultivate for 4 h, the medium was discarded and 150 μL DMSO was added. The 96-well plate was taken out and was left to stand for 1 min. Then the 96-well plate was placed in the microplate reader, the absorbance values of each well were measured at 490 nm, and the reactive oxygen species were calculated in each group. IL-8, TNF-α, and IL-1β in the supernatant of GES-1 cells in each group were analyzed by enzyme-linked immunosorbent assay (ELISA) [59]. The ROS of cells in each group were analyzed using the dichloro-dihydro-fluorescein diacetate (DCFH-DA) method.

#### 4.9.3. Apoptosis Analysis by Flow Cytometry

Cells were digested routinely and seeded in a 6-well plate, including 2.0 mL medium·well^−1^. The cells in each group were collected for detection after 8 h of cultivation according to the groups described above. Double-distilled water was used to dilute the 5× binding buffer. The binding buffer was diluted as follows: 1× binding Buffer in 0.5 mL final volume. We mixed 5.0 μL Annexin V and 10.0 μL propidium iodide (PI) to prepare the Annexin V/PI dye working solution. The old cell culture medium was discarded and 2.0 mL phosphate-buffered saline (PBS)/well was added to gently rinse the cells in the culture plate. The PBS was then removed, 1.0 mL of 0.25% trypsin was added into each well and the plate was incubated. Under the microscope, when the cells were loose, 2.0 mL PBS was added to prepare the single cell suspension. The Annexin V/PI dye working solution (200 μL) was then added, cells were re-suspended, blown gently, and incubated in the dark for 15 min before detection by flow cytometer [60].

### 4.10. Statistical Analysis

The test results were expressed as mean ± standard deviation (SD). The statistical analysis was conducted using SPSS 23.0 and all data analysis of variance (ANOVA) was carried out using the LSD test.

## 5. Conclusions

In this experiment, an active polysaccharide (VUP80-3) was extracted from *V. unguiculata*. The main chain of VUP80-3 may be →4)-α-D-Glcp(1→ and →4)-α-D-Galp(1→, the branch chain may be →3,4)-β-D-Glcp(1→, →4,6)-α-D-Glcp(1→, and sugar terminated α-D-Glcp(1→. The biological activity test showed that VUP80-3 performed well in vitro to repair damaged gastric mucosal cells. The results suggested that 1000 μg·mL^−1^ of VUP80-3 could significantly increase the activity of ethanol injured GES-1 cells (*p* < 0.01), significantly reduce the levels of ROS and inflammatory factors in ethanol injured GES-1 cells (*p* < 0.05) and reduce the rate of apoptosis (*p* < 0.05). Therefore, this polysaccharide significantly reduced the oxidative damage caused by ethanol in GES-1 cells. These data suggest that VUP80-3 is a potential drug that can be used to protect the gastric mucosa from damage. However, does VUP80-3 improve inflammatory factors in gastric mucosal cells by reducing ROS in cells? What is the specific role of decreased apoptosis rate in inhibiting gastric mucosal injury? Is there a relationship between mucosal secretion and alcohol exposure on the gene level? Furthermore, the signal pathway of VUP80-3 protecting gastric mucosal cells needs further study.

## Figures and Tables

**Table 1:** Results of the methylation analysis.

Linkage Type	Methylation Product	Molecular Weight (M_W_)	RT (min)	Main MS (*m/z*)
t-Ara(f)	1,4-di-O-acetyl-2,3,5-tri-O-methyl arabinitol	279	5.71	71, 87, 101, 118, 129, 161
t-Glc(p)	1,5-di-O-acetyl-2,3,4,6-tetra-O-methyl glucitol	323	8.58	59, 71, 87, 102, 118, 129, 145, 162, 205
t-Gal(p)	1,5-di-O-acetyl-2,3,4,6-tetra-O-methyl galactitol	323	9.57	59, 71, 87, 102, 118, 129, 145, 157, 205
5-Ara(f)	1,4,5-tri-O-acetyl-2,3-di-O-methyl arabinitol	307	10.21	71, 87, 102, 118, 129, 189
2,4-Rha(p)	1,2,4,5-tetra-O-acetyl-6-deoxy-3-O-methyl rhamnitol	349	12.09	59, 74, 88, 101, 130, 143, 190, 203
4-Gal(p)	1,4,5-tri-O-acetyl-2,3,6-tri-O-methyl galactitol	351	13.32	71, 87, 99, 118, 131, 142, 157, 173, 233
4-Glc(p)	1,4,5-tri-O-acetyl-2,3,6-tri-O-methyl glucitol	351	13.65	71, 87, 99, 118, 129, 142, 157, 173, 233
3,4-Glc(p)	1,3,4,5-tetra-O-acetyl-2,6-di-O-methyl glucitol	379	15.82	59, 87, 118, 129, 160, 171, 185, 305
2,4-Gal(p)	1,2,4,5-tetra-O-acetyl-3,6-di-O-methyl galactitol	379	16.47	59, 71, 87, 99, 113, 130, 190, 233
4,6-Glc(p)	1,4,5,6-tetra-O-acetyl-2,3-di-O-methyl glucitol	379	17.88	59, 85, 101, 117, 159, 261

**Table 2:** Chemical shifts of sugar residues ^1^H and ^13^C.

Code	Glycosyl Residues	Chemical Shifts (ppm)					
		H1/C1	H2/C2	H3/C3	H4/C4	H5/C5	H6a,b/C6
A	→4)-α-D-Glcp(1→	5.42	3.65	3.99	3.69	3.85	3.84/n.d
		102.45	74.38	76.1	79.46	74.03	63.24
B	→4)-α-D-Galp(1→	4.99	3.78	3.92	3.65	3.87	3.85/n.d
		100.54	70.71	72.99	74.19	73.83	63.2
C	α-D-Glcp(1→	4.66	3.7	3.85	3.43	3.86	3.65/n.d
		107.22	74.7	74.04	72.11	74.06	60.24
D	→3,4)-β-D-Glcp(1→	5.1	3.54	4.14	4.02	3.97	3.79/n.d
		110.36	72.27	83.85	79.36	76.26	63.62
E	→4,6)-α-D-Glcp(1→	5.1	3.59	3.96	3.73	3.84	3.9/n.d
		101.86	74.21	75.54	79.49	76.19	69.67
F	→5)-α-L-Araf(1→	5.79	4.26	4.19	n.d	3.77,4.01	-
		109.61	85.17	80.3	n.d	68.27	-

**Table 3:** Effect of VUP80-3 on the secretion of inflammatory cytokines by GES-1 injured cells.

Inflammatory Factors (pg/mg Prot)	Groups (μg/mL)				
	Control	Model	250	500	1000
IL-8	48.11 ± 2.78 **	125.6 ± 3.85	101.99 ± 6.38	82.75 ± 5.07 *	68.05 ± 4.31 **
TNF-α	15.95 ± 4.74 **	58.19 ± 2.69	46.91 ± 3.73	33.16 ± 3.48	25.32 ± 2.68 *
IL-1β	18.96 ± 2.77 **	46.34 ± 3.07	33.38 ± 1.99	28.57 ± 2.07 *	19.58 ± 1.79 **

Note: Data were expressed as mean ± SD from three independent experiments. * *p* < 0.05 and ** *p* < 0.01 when compared with the model group.

## Data Availability

Not applicable.

## Supplementary Materials

The following supporting information can be downloaded at: https://www.mdpi.com/article/10.3390/molecules28145566/s1, Figure S1: GPC analysis of VUP80-3; Figure S2: TIC chromatogram of VUP80-3.

## References

[1] Czekaj R., Majka J., Magierowska K., Sliwowski Z., Magierowski M., Pajdo R., Brzozowski T. (2017). Mechanisms of curcumin-induced gastroprotection against ethanol-induced gastric mucosal lesions. J. Gastroenterol..

[2] Meng J., Chen T., Zhao Y., Lu S., Yu H., Chang Y., Chen D. (2019). Study of the mechanism of anti-ulcer effects of virgin coconut oil on gastric ulcer-induced rat model. Arch. Med. Sci..

[3] Chang W., Bai J., Tian S., Ma M., Li W., Yin Y., Deng R., Cui J., Li J., Wang G. (2017). Autophagy protects gastric mucosal epithelial cells from ethanol-induced oxidative damage via mTOR signaling pathway. Exp. Biol. Med..

[4] Gong H., Li W., Sun J., Jia L., Guan Q., Guo Y., Wang Y. (2022). A review on plant polysaccharide based on drug delivery system for construction and application, with emphasis on traditional Chinese medicine polysaccharide. Int. J. Biol. Macromol..

[5] Shen C., Wang T., Guo F., Sun K., Wang B., Wang J., Zhang Z., Zhang X., Zhao Y., Chen Y. (2021). Structural characterization and intestinal protection activity of polysaccharides from Sea buckthorn (*Hippophae rhamnoides* L.) berries. Carbohydr. Polym..

[6] Liu Y., Sui D., Fu W., Sun L., Li Y., Yu P., Yu X., Zhou Y., Xu H. (2021). Protective effects of polysaccharides from *Panax ginseng* on acute gastric ulcers induced by ethanol in rats. Food Funct..

[7] Ranade A.N., Wankhede S.S., Ranpise N.S., Mundada M.S. (2012). Development of bilayer floating tablet of amoxicillin and *Aloe vera* gel powder fortreatment of gastric ulcers. AAPS Pharm. Sci. Tech..

[8] Subramanian S., Sathish Kumar D., Arulselvan P., Senthilkumar G.P., Mahadeva Rao U.S. (2007). Evaluation of anti-ulcerogenic potential of *Aloe vera* leaf gel extract studied in experimental rats. J. Pharmacol. Toxicol..

[9] Metowogo K., Eklu-Gadegbeku K., Agbonon A., Aklikokou K.A., Gbeassor M. (2011). Gastroprotective effect of hydroalcoholic extract of Aloe buettneri. Iran. J. Pharm. Res..

[10] Wang Y., Su W., Zhang C., Xue C., Chang Y., Wu X., Tang Q., Wang J. (2012). Protective effect of sea cucumber (*Acaudina molpadioides*) fucoidan against ethanol-induced gastric damage. Food Chem..

[11] Kan J., Hood M., Burns C., Scholten J., Chuang J., Tian F., Pan X., Du J., Gui M. (2017). A Novel Combination of Wheat Peptides and Fucoidan Attenuates Ethanol-Induced Gastric Mucosal Damage through Anti-Oxidant, Anti-Inflammatory, and Pro-Survival Mechanisms. Nutrients.

[12] Chen W., Wu D., Jin Y., Li Q., Liu Y., Qiao X., Zhang J., Dong G., Li Z., Li T. (2020). Pre-protective effect of polysaccharides purified from *Hericium erinaceus* against ethanol-induced gastric mucosal injury in rats. Int. J. Biol. Macromol..

[13] Li A.L., Xiong S.L. (2008). Evaluation of amino acid content and nutritional value of cowpea seed protein. Food Res. Dev..

[14] Abd-Alla M.H., Bashandy S.R., Nafady N.A., Hassan A.A. (2018). Enhancement of exopolysaccharide production by *Stenotrophomonas maltophilia* and *Brevibacillus parabrevis* isolated from root nodules of *Cicer arietinum* L. and *Vigna unguiculata* L. (Walp.) plants, *Rendiconti lincei*. Sci. Fis. E Nat..

[15] Moloto M.R., Phan A.D.T., Shai J.L., Sultanbawa Y., Sivakumar D. (2020). Comparison of Phenolic Compounds, Carotenoids, Amino Acid Composition, In Vitro Antioxidant and Anti-Diabetic Activities in the Leaves of Seven Cowpea (*Vigna unguiculata*) Cultivars. Foods.

[16] Rengadu D., Gerrano A.S., Mellem J.J. (2020). Physicochemical and structural characterization of resistant starch isolated from *Vigna unguiculata*. Int. J. Biol. Macromol..

[17] Xin Y., Song X., Wang Y., Nie S., Yin J. (2022). Structural Characteristics and Antioxidant Properties of Polysaccharides from Different Parts of Cowpea. Food Biosci..

[18] Zhang B., Deng Z., Ramdath D.D., Tang Y., Chen P.X., Liu R., Liu Q., Tsao R. (2015). Phenolic profiles of 20 Canadian lentil cultivars and their contribution to antioxidant activity and inhibitory effects on α-glucosidase and pancreatic lipase. Food Chem..

[19] Wang Z., Zhou X., Shu Z., Zheng Y., Hu X., Zhang P., Huang H., Sheng L., Zhang P., Wang Q. (2023). Regulation strategy, bioactivity, and physical property of plant and microbial polysaccharides based on molecular weight. Int. J. Biol. Macromol..

[20] Luo A., Ge Z., Fan Y., Luo A., Chun Z., He X. (2011). In Vitro and In Vivo Antioxidant Activity of a Water-Soluble Polysaccharide from *Dendrobium denneanum*. Molecules.

[21] Fan Y., Ma J., Wang G., Li X., Liu Y., Xu E., Luo A. (2023). Ultrasonic extraction, structural modification and gastric mucosal cells protective activity of a polysaccharide from *Dendrobium denneanum*. Arab. J. Chem..

[22] Fan Y., Lin M., Luo A., Chun Z., Luo A. (2014). Characterization and Antitumor Activity of a Polysaccharide from *Sarcodia ceylonensis*. Molecules.

[23] Fan Y., He Q., Luo A., Wang M., Luo A. (2015). Characterization and Antihyperglycemic Activity of a Polysaccharide from *Dioscorea opposita* Thunb Roots. Int. J. Mol. Sci..

[24] Wang W., Li X., Chen K., Yang H., Jialengbieke B., Hu X. (2020). Extraction optimization, characterization and the antioxidant activities in vitro and in vivo of polysaccharide from Pleurotus ferulae. Int. J. Biol. Macromol..

[25] Miao M., Bai A., Jiang B., Song Y., Cui S.W., Zhang T. (2014). Characterisation of a novel water-soluble polysaccharide from leuconostoc citreum SK24.002. Food Hydrocoll..

[26] Fan Y., Lin M., Luo A. (2021). Extraction, characterization and antioxidant activities of an acidic polysaccharide from *Dendrobium devonianum*. J. Food Meas. Charact..

[27] Sims I.M., Carnachan S.M., Bell T.J., Hinkley S.F. (2018). Methylation analysis of polysaccharides: Technical advice. Carbohydr. Polym..

[28] Li J., Zhong Y.G., Liu C.J. (2010). Purification and Electron Microscope Analysis of Lentinan. J. Shanxi Agric. Sci..

[29] Li G.H., Wang J., Gao X.L., Guo W.Y., Zhang Y.L., Wang Y.H., Hou Y.F. (2019). Effect of ultrafine grinding of cowpea powder on its physicochemical properties and antioxidant activity. Food Sci. Technol..

[30] Zeng Q., Ko C.H., Siu W.S., Li L.F., Han X.Q., Yang L., Lau C.B.S., Hu J.M., Leung P.C. (2017). Polysaccharides of Dendrobium officinale Kimura & Migo protect gastric mucosal cell against oxidative damage-induced apoptosis in vitro and in vivo. J. Ethnopharmacol..

[31] Zhou D., Yang Q., Tian T., Chang Y., Li Y., Duan L.R., Li H., Wang S.W. (2020). Gastroprotective effect of gallic acid against ethanol-induced gastric ulcer in rats: Involvement of the Nrf2/HO-1 signaling and anti-apoptosis role. Biomed. Pharmacother..

[32] Beiranvand M., Bahramikia S. (2020). Ameliorating and protective effects mesalazine on ethanol-induced gastric ulcers in experimental rats. Eur. J. Pharmacol..

[33] Amirshahrokhi K., Khalili A.-R. (2015). The effect of thalidomide on ethanol-induced gastric mucosal damage in mice: Involvement of inflammatory cytokines and nitric oxide. Chem. Interact..

[34] Konturek P.C., Duda A., Brzozowski T., Konturek S.J., Kwiecien S., Drozdowicz D., Pajdo R., Meixner H., Hahn E.G. (2000). Activation of genes for superoxide dismutase, interleukin-1beta, tumor necrosis factor-alpha, and intercellular adhesion molecule-1 during healing of ischemia-reperfusion-induced gastric injury. Scand. J. Gastroenterol..

[35] Yang X., Yang L., Pan D., Liu H., Xia H., Wang S., Sun G. (2020). Wheat peptide protects against ethanol-induced gastric mucosal damage through downregulation of TLR4 and MAPK. J. Funct. Foods.

[36] Fujiwara Y., Arakawa T., Fukuda T., Sasaki E., Nakagawa K., Fujiwara K., Higuchi K., Kobayashi K., Tarnawski A. (1997). Interleukin-8 stimulates leukocyte migration across a monolayer of cultured rabbit gastric epithelial cells. Effect associated with the impairment of gastric epithelial barrier function. Dig. Dis. Sci..

[37] Watanabe T., Higuchi K., Tanigawa T., Tominaga K., Fujiwara Y., Arakawa T. (2022). Mechanisms of peptic ulcer recurrence: Role of inflammation. Inflammopharmacology.

[38] De Araújo E.R.D., Guerra G.C.B., Araújo D.F.d.S., De Araújo A.A., Fernandes J.M., De Araújo Júnior R.F., Da Silva V.C., De Carvalho T.G., Ferreira L.D.S., Zucolotto S.M. (2018). Gastroprotective and Antioxidant Activity of *Kalanchoe brasiliensis* and *Kalanchoe pinnata* Leaf Juices against Indomethacin and Ethanol-Induced Gastric Lesions in Rats. Int. J. Mol. Sci..

[39] Yang K., Zhan L.H., Lu T.T., Zhou C., Chen X., Dong Y.J., Lv G.Y., Chen S.H. (2020). Polysaccharides of *Dendrobium officinale* Kimura & Migo Leaves Protect Against Ethanol-Induced Gastric Mucosal Injury via the AMPK/mTOR Signaling Pathway in Vitro and vivo. Front. Pharmacol..

[40] Nam H.H., Choo B.K. (2021). Geranium koreanum, a medicinal plant Geranii Herba, ameliorate the gastric mucosal injury in gastritis-induced mice. J. Ethnopharmacol..

[41] Dumitriu S. (2005). Polysaccharides: Structural Diversity and Functional Versatility.

[42] Ju H., Yu C., Zhang X.D., Liu W., Wu Y.C., Gong P.X., Li H.H., Liu Y., Li H.J. (2023). Recent trends in anti-cancer activities of terrestrial plants-based polysaccharides: A review. Carbohydr. Polym. Technol. Appl..

[43] Liao H.F., Chen Y., Yang Y. (2005). A novel polysaccharide of black soybean promotes myelopoiesis and reconstitutes bone marrow after 5-flurouraciland irradiation-induced myelosuppression. Life Sci..

[44] Wu M.-H., Lee Y.-C., Tsai W.-J., Yang W.-B., Chen Y.-C., Chuang K.-A., Liao J.-F., Wang C.-C., Kuo Y.-C. (2010). Characterized Polysaccharides from Black Soybean Induce Granulocyte Colony-Stimulated Factor Gene Expression in a Phosphoinositide 3-Kinase-dependent Manner. Immunol. Investig..

[45] Yao Y., Hu G., Gao Y., Wen Z. (2012). White lentil polysaccharide inhibits hypoxic necrosis and apoptosis of nerve cells. Pharmacol. Clin. Appl. Tradit. Chin. Med..

[46] Campos-Vega R., Guevara-Gonzalez R., Guevara-Olvera B., Oomah B.D., Loarca-Piña G. (2010). Bean (*Phaseolus vulgaris* L.) polysaccharides modulate gene expression in human colon cancer cells (HT-29). Food Res. Int..

[47] Cheng A., Wu J., Qin H., Yang Q., Liu C., Guo X., Sun J. (2017). Study on the content and antioxidant activity of polyphenols and flavonoids in four types of legumes. Chin. J. Cereals Oils.

[48] Wu G.-J., Liu D., Wan Y.-J., Huang X.-J., Nie S.-P. (2018). Comparison of hypoglycemic effects of polysaccharides from four legume species. Food Hydrocoll..

[49] Lai F., Wen Q., Li L., Wu H., Li X. (2010). Antioxidant activities of water-soluble polysaccharide extracted from mung bean (*Vigna radiata* L.) hull with ultrasonic assisted treatment. Carbohydr. Polym..

[50] Qiang Y., Zhu W., Zhihan G., Xin Q., Siyu W., Tuoping L., Suhong L. (2021). Optimization of Ultrasound Assisted Extraction of Cowpea Polysaccharides and in Vitro Antioxidant Study. J. Shenyang Agric. Univ..

[51] Luo A., Fan Y., Tan X., Zhao J., Yang K., Wu S., Zhang J., Pu S., Wang G. (2022). Screening and characterization of an acid polysaccharide with antioxidant activity in vitro and in vivo from *Dendrobium aurantiacum* var. *denneanum* (Kerr). Pharmacogn. Mag..

[52] Fan Y., He X., Zhou S., Luo A., He T., Chun Z. (2009). Composition analysis and antioxidant activity of polysaccharide from *Dendrobium denneanum*. Int. J. Biol. Macromol..

[53] Luo A., He X., Zhou S., Fan Y., Luo A., Chun Z. (2010). Purification, composition analysis and antioxidant activity of the polysaccharides from *Dendrobium nobile* Lindl. Carbohydr. Polym..

[54] Luo A., He X., Zhou S., Fan Y., He T., Chun Z. (2009). In vitro antioxidant activities of a water-soluble polysaccharide derived from Dendrobium nobile Lindl. extracts. Int. J. Biol. Macromol..

[55] Xu J., Wang R., Liu J., Cheng H., Yu N. (2021). Determination of monosaccharides in *Lycium barbarum* fruit polysaccharide by an efficient UHPLC-QTRAP-MS/MS method. Phytochem. Anal..

[56] Liu W., Li Z., Feng C., Hu S., Yang X., Xiao K., Nong Q., Xiao Q., Wu K., Li X.Q. (2022). The structures of two polysaccharides from *Angelica sinensis* and their effects on hepatic insulin resistance through blocking RAGE. Carbohydr. Polym..

[57] Wan X., Jin X., Wu X., Yang X., Lin D., Li C., Fu Y., Liu Y., Liu X., Lv J. (2021). Structural characterisation and antitumor activity against non-small cell lung cancer of polysaccharides from *Sanghuangporus vaninii*. Carbohydr. Polym..

[58] Fan Y., Yu Q., Wang G., Tan J., Liu S., Pu S., Chen W., Xie P., Zhang Y., Zhang J. (2020). Effects of Non-thermal Plasma Treatment on the Polysaccharide from *Dendrobium nobile* Lindl. and Its Immune Activities in vitro. Int. J. Biol. Macromol..

[59] Quan Z., Guan R., Huang H., Yang K., Cai M., Meng X. (2020). Antioxidant activity and absorption of cyanidin-3-O-glucoside liposomes in GES-1 cells in vitro. Biosci. Biotechnol. Biochem..

[60] Hany H.A., Ahmed M.A., Amany M.G., Ayman M.M., Ahmed M.K. (2021). Activation of AMPK/mTOR-driven autophagy and inhibition of NLRP3 inflammasome by saxagliptin ameliorate ethanol-induced gastric mucosal damage. Life Sci..

