# Supplementary figures and images for: Isolation, Structural Characteristics Analysis of a Vigna unguiculata Polysaccharide VUP80-3 and Its Protective Effect on GES-1 Cells In Vitro
Source: Molecules. 2023 Jul 21;28(14):5566. doi: 10.3390/molecules28145566 (PMC10383257; doi:10.3390/molecules28145566)

## Supplementary data

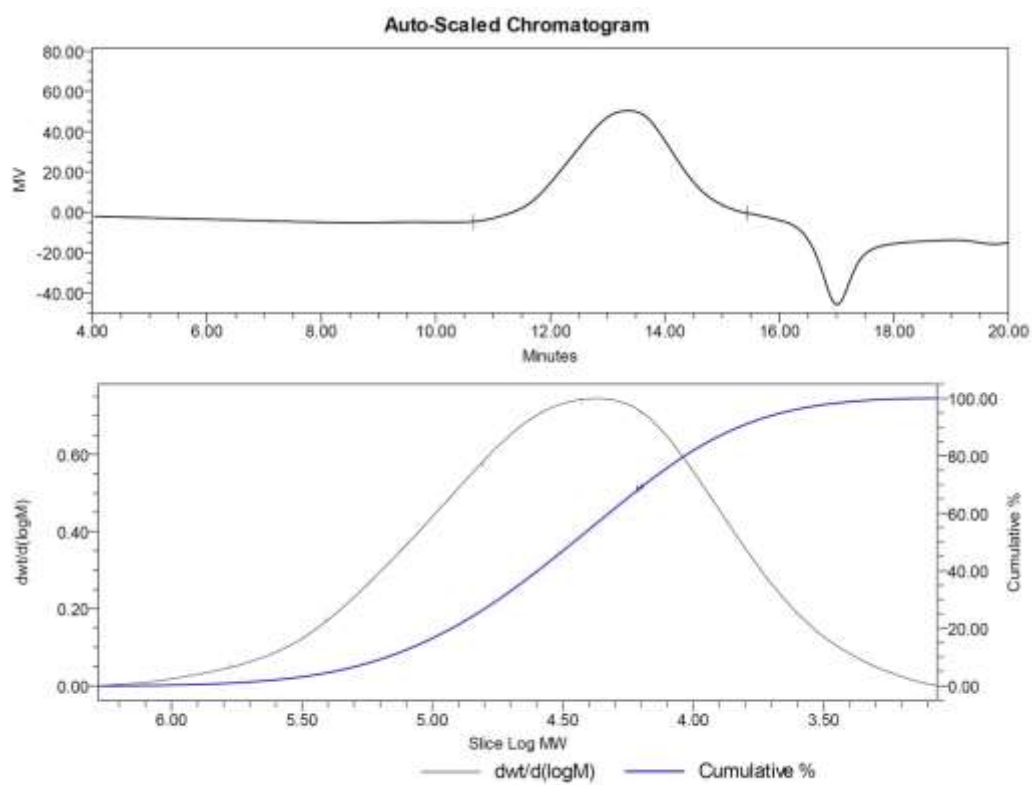

Figure S1. GPC analysis of VUP80-3

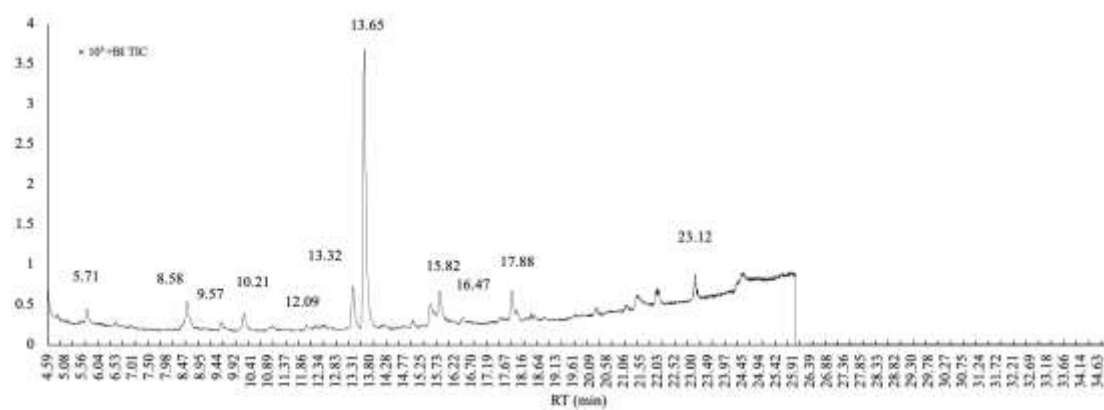

Figure S2. TIC chromatogram of VUP80-3

Supplement: Supplementary file 1 [file molecules-28-05566-s001.zip › molecules-2518834-supplementary.pdf]
